# Addressing indirect frequency coupling via partial generalized coherence

**DOI:** 10.1038/s41598-021-85677-6

**Published:** 2021-03-22

**Authors:** Joseph Young, Ryota Homma, Behnaam Aazhang

**Affiliations:** 1 Department of Electrical and Computer Engineering, Rice University, Houston, TX 77005 USA; 2 Department of Neurobiology and Anatomy, McGovern Medical School at the University of Texas Health Science Center at Houston, Houston, 77030 USA

**Keywords:** Olfactory bulb, Neuroscience, Computational neuroscience

## Abstract

Distinguishing between direct and indirect frequency coupling is an important aspect of functional connectivity analyses because this distinction can determine if two brain regions are directly connected. Although partial coherence quantifies partial frequency coupling in the linear Gaussian case, we introduce a general framework that can address even the nonlinear and non-Gaussian case. Our technique, partial generalized coherence (PGC), expands prior work by allowing pairwise frequency coupling analyses to be conditioned on other processes, enabling model-free partial frequency coupling results. By taking advantage of recent advances in conditional mutual information estimation, we are able to implement our technique in a way that scales well with dimensionality, making it possible to condition on many processes and produce a partial frequency coupling graph. We analyzed both linear Gaussian and nonlinear simulated networks. We then performed PGC analysis of calcium recordings from mouse olfactory bulb glomeruli under anesthesia and quantified the dominant influence of breathing-related activity on the pairwise relationships between glomeruli for breathing-related frequencies. Overall, we introduce a technique capable of eliminating indirect frequency coupling in a model-free way, empowering future research to correct for potentially misleading frequency interactions in functional connectivity analyses.

## Introduction

Functional connectivity analyses, which consider statistical relationships between time series recorded from brain regions or substructures^1^, have been crucial for advances in neuroscience^2^. The variety of techniques to quantify functional connectivity that exist can be differentiated by whether they consider relationships in the time domain or the frequency domain^3^, the latter of which is the focus of this work. Measures of functional connectivity in the frequency domain, i.e. frequency coupling (FC), have been tremendously helpful in shedding light on issues including Alzheimer’s^4^, cognition^5^, memory^6^, and epilepsy^7^. Coherence is perhaps the most common approach to measuring same-frequency coupling resulting from linear interactions and specifically quantifies correlation in the frequency domain^8–10^. Cross-frequency coupling (CFC) metrics attempt to alleviate the linear same-frequency restriction of coherence by considering interactions between spectral components of different processes at different frequencies^11^.

However, these methods fall short of quantifying full statistical dependence in the frequency domain. Critically, correlation-based functional connectivity metrics cannot fully describe non-Gaussian^12^ or nonlinear^13^ neural activity. For example, coherence or any correlation-based CFC metric will only be a full measure of statistical dependence for the Gaussian case. In light of the failure of existing methods to comprehensively quantify statistical dependence, Mutual Information (MI) in frequency was introduced^7,14^ and drew inspiration from prior work on same-frequency coupling^15^. Remarkably, MI in frequency (MIF) makes no model assumptions and therefore measures statistical dependence between any types of processes. It can be applied to processes with nonlinear relationships which result in CFC, overcoming the same-frequency limitation of coherence.

Although the capabilities of MIF are notable, one has still lacked a model-free way to distinguish between direct and indirect frequency coupling which is ambiguously identified by pairwise methods such as MIF. Such a distinction is critical in neuroscience since it is desirable to know if two brain regions or substructures are uniquely related or only have a proxy relationship. Assuming the linear Gaussian case, the partial coherence technique^8,9,16^ is readily available and well-studied, acting as an analog to the partial correlation in the frequency domain. Beyond this, however, this is no general technique that has been introduced to deal with the non-Gaussian and nonlinear scenario that is most relevant for neural activity.

Therefore, we introduce a new functional connectivity technique, partial generalized coherence (PGC), which is a partial expansion of the MIF^7^ technique that it itself could be regarded as generalized coherence. By leveraging the model-free capabilities of conditional MI we are able to infer and eliminate indirect frequency coupling by conditioning pairwise MIF analyses on the spectral components of other processes without making any model assumptions. Furthermore, we integrate recent advances in conditional MI estimation^17^ that allow for PGC to condition on a significant number of other spectral components of other processes. This enhanced scaling performance ultimately enables the estimation of model-free partial frequency coupling graphs, where edges would be quantified by PGC.

Beyond demonstration of PGC’s scalable conditioning abilities in a variety of simulations, an intriguing experimental application for our technique is the quantification of the effect of breathing activity on glomerular relationships within the rodent olfactory bulb (OB)^18^. The OB is the fundamental site of early olfactory processing, which receives inputs from olfactory sensory neurons that interact directly with odorants. Such inputs from sensory neurons are converged at sites called glomeruli and then transmitted via other cells to cortical sites for further processing, leading to odor perception. Short-axon cells^19–21^ are known to mediate connections between glomeruli, however quantification of glomerular interactions remains severely underexplored. Since it is clear that breathing modulates glomerular activity^22^, we employ PGC to explore how glomerular CFC is altered by conditioning on the spectral components of the breathing signal.

The rest of this manuscript is divided as follows. In the methods section we first provide context by reviewing partial coherence. Then we introduce PGC which acts as a generalization of partial coherence, and we follow with a graph extension of PGC. To close the methods section, we explain how one estimates PGC from data. We begin our results section with an intuitive Gaussian process example. We then show the potential of PGC for much more complex problems with a nonlinear and non-Gaussian example. Scaling performance is then discussed in order to show the ability of PGC to condition on many nodes. Finally, we use PGC on glomerular calcium data from the rodent OB in order to explore the extent to which breathing activity dominates pairwise glomerular relationships at breathing-related frequencies.

**Figure 1:**
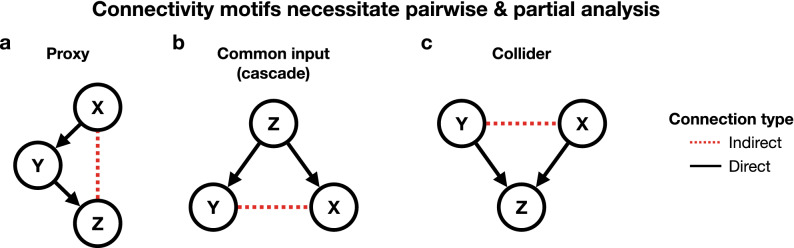
Overview of connectivity motifs^23–25^ that can lead to incorrect inference without pairwise and partial analysis. Arrows indicate variable definitions. For example, X$\rightarrow$Y denotes that Y is generated by adding noise to X, while X is independently generated. (**a**) X and Z will exhibit a non-zero pairwise relationship, however controlling for Y will eliminate this indirect relationship. (**b**) Y and X will exhibit a non-zero pairwise relationship, however controlling for Z will eliminate this indirect relationship. (**c**) When Y and X are independent, a partial analysis between them conditioned on Z will produce a non-zero value.

## Methods

### Direct and indirect relationships

It is critical in neuroscience to distinguish between direct and indirect relationships as this corresponds to whether or not brain regions are directly functionally connected. Functional connectivity in the frequency domain is the primary focus of this work, i.e. distinguishing between direct and indirect frequency coupling. When multiple channels of data are being analyzed, there are a few particular network motifs^23–25^ that need to be addressed in order to identify direct connections (Fig. 1). Note that in Fig. 1 arrows indicate how variables are generated. For example, X$\rightarrow$Y signifies that Y was generated by adding noise to X, while X was generated independently. These arrows are particularly important in being able to distinguish between the motifs of Fig. 1b,c, which would otherwise appear identical despite being quite different. We emphasize that arrows do not denote causality or directionality in relationships. The first of the motifs, a *proxy*^23^ (Fig. 1a) connection, occurs when three nodes have a sequential connection structure, causing pairwise methods to incorrectly identify a connection between the first and final nodes. A *common input* or *cascade*^23^ (Fig. 1b) configuration occurs when one node serves as an input to two other nodes, inducing an indirect connection between the two other nodes that will be mistakenly identified in pairwise inference. When two nodes influence a third node, i.e. a *collider*^25^ (Fig. 1c), then a partial analysis between the first two nodes conditioned on the third node will produce a non-zero value despite the underlying independence between those two nodes.

In light of the possible network motifs (Fig. 1), both pairwise and partial methods must be used together^25^ in order to correctly identify direct frequency coupling between nodes. Although partial methods which condition analyses on other nodes can eliminate indirect connections in the proxy and common input scenarios (Fig. 1a,b), pairwise methods are needed to identify collider scenarios (Fig. 1c) since such methods will identify the two source nodes as independent^25^. Therefore, our PGC framework will include insight from the pairwise method MIF in order to address the collider motif while otherwise automatically addressing the proxy and common input motifs.

### Partial coherence

A common method for quantifying frequency coupling in functional connectivity analyses is coherence, which serves as a frequency domain version of the correlation coefficient^8,9^. Consider two Gaussian processes *X*(*t*) and *Y*(*t*) which have power spectral densities $S_{X}(f)$ and $S_{Y}(f)$, respectively, as well as the cross power spectral density $S_{XY}(f)$. Assuming the processes are jointly and individually wide sense stationary, coherence can be defined as^8,9^:

1 $$\begin{aligned} C_{XY}(f) = \frac{|S_{XY} (f)|^2}{S_{X} (f)S_{Y} (f)}. \end{aligned}$$

We note that this definition of coherence has the advantage of being real-valued and ranging from 0 to 1, where 0 indicates no correlation between *X*(*t*) and *Y*(*t*) for a particular frequency whereas 1 indicates maximum correlation at that frequency^8^.

However, coherence only considers frequency correlations between two processes, meaning that indirect and direct frequency coupling will be indistinguishable from each other which leaves analyses susceptible to particular network motifs (Fig. 1). In order to solve this problem for Gaussian processes, one naturally considers the partial coherence^8,9^. Partial coherence captures the unique frequency correlations between two processes, as it is effectively a frequency version of the partial correlation^8^. The partial coherence for three processes is computed by first constructing a matrix ${\mathbf {S}}(f)$ containing the power spectral densities for processes *X*(*t*), *Y*(*t*), and *Z*(*t*) on its diagonal, and the appropriate cross power spectral densities on its off-diagonal^8^:

2 $$\begin{aligned} {\mathbf {S}}(f) = \begin{bmatrix} S_X(f) &{} S_{XY}(f) &{} S_{XZ}(f) \\ S_{XY}(f) &{} S_Y(f) &{} S_{YZ}(f) \\ S_{XZ}(f) &{} S_{YZ}(f) &{} S_Z(f) \end{bmatrix}. \end{aligned}$$

Considering the inverse of this matrix ${\mathbf {P}}(f)={\mathbf {S}}(f)^{-1}$, with elements $P_{X}(f), P_{XY}(f),\dots , P_Y(f)$, the partial coherence between *X*(*t*) and *Y*(*t*) at frequency *f* is^8,9^:

3 $$\begin{aligned} C_{XY|Z}(f) = \frac{|P_{XY} (f)|^2}{P_{X} (f)P_{Y} (f)}. \end{aligned}$$

Similar to coherence, $C_{XY|Z}(f)$ is real-valued and ranges from 0 to 1^9^, however now it provides the coherence between *X*(*t*) and *Y*(*t*) for a particular frequency with the linear influence of *Z*(*t*) eliminated^26^.

We emphasize that coherence and partial coherence are only measures of full statistical dependence in the case of Gaussian processes. This is because both techniques rely on second-order statistics, which are only sufficient descriptions of the relationships between Gaussian processes. For measuring the statistical dependence between spectral components of random processes in general, one must consider a more advanced approach.

### Partial generalized coherence (PGC)

While coherence and partial coherence are sufficient frequency coupling tools only for linear Gaussian processes, MIF^7,27^, which drew from same-frequency coupling work^15^, is a general technique with no model assumptions quantifying CFC. Although polyspectral methods^28,29^ can capture more general phenomena than coherence and partial coherence, such methods will still fall short of quantifying the full statistical dependence without model assumptions that MIF quantifies because of polyspectral methods’ general reliance on the expectation of a product, which is similar to a correlation and restrictive. By contrast, MIF relies on spectral probability distributions and therefore doesn’t assume any model about the interaction between processes.

Considering a random process *X*(*t*) which no longer has to be a Gaussian process, we can define the Cramér representation of this process which provides a time-frequency relationship and also define the process’s particular frequency domain representation^7,30,31^:

4 $$\begin{aligned} X(t) = \int _{-\infty }^{\infty }e^{j2\pi f t}d{\tilde{X}}(f), \quad {\tilde{X}}(f) = {\tilde{X}}_R(f) + j{\tilde{X}}_I(f), \end{aligned}$$

with the integral being a Fourier–Stieltjes integral and $d{\tilde{X}}(f)$ denoting a spectral increment. Although $d{\tilde{X}}(f) = d{\tilde{X}}_R(f) + jd{\tilde{X}}_I(f)$, we change this definition for convenience to a two-dimensional vector, i.e. $d{\tilde{X}}(f)=[d{\tilde{X}}_R(f),\,d{\tilde{X}}_I(f)]$.

Consider MI, which is a generalization of correlation that quantifies the statistical dependence between any two random variables. Formally, the MI between random variables *X* and *Y* with associated marginal probability densities $p_X(x)$ and $p_Y(y)$ as well as joint density $p_{XY}(x,y)$ is^32^:

5 $$\begin{aligned} I(X;Y)&= D_{KL}(p_{X,Y}\,||\,p_Xp_Y) \end{aligned}$$

6 $$\begin{aligned}{}&= \int _{\mathbb {Y}}\int _{\mathbb {X}} p_{X,Y}(x,y)\log \bigg (\frac{p_{X,Y}(x,y)}{p_X(x)p_Y(y)}\bigg )dxdy, \end{aligned}$$

where $D_{KL}$ is the Kullback–Leibler (KL) divergence quantifying the difference between the marginal and joint densities, while $\mathbb {X}$ and $\mathbb {Y}$ denote the supports of *X* and *Y* where $p_X(x)>0$ and $p_Y(y)>0$. The intuition behind MI is that it uses the classical definition of statistical independence, $p_{XY}(x,y)=p_X(x)p_Y(y)$, as a ratio. When *X* and *Y* are independent, the ratio is 1 and therefore MI will be 0 as well. Any deviation from statistical independence will be accordingly measured by MI. It is precisely this use of probabilities that allows MI to capture non-Gaussian and nonlinear relationships.

The MIF between two random processes *X*(*t*) and *Y*(*t*) is therefore the MI between spectral increments of each process at particular frequencies^7^:

7 $$\begin{aligned} MI_{XY}(f_i,f_j) = I(d{\tilde{X}}(f_i);d{\tilde{Y}}(f_j)). \end{aligned}$$

For the particular case where *X*(*t*) and *Y*(*t*) are Gaussian processes, the MIF between them can be directly related to their coherence^7^:

8 $$\begin{aligned} MI_{XY}(f_i,f_i)=-\log (1-C_{XY}(f_i)), \end{aligned}$$

which was proven explicitly^7^ and is related to previous work^15,33^. Note that $MI_{XY}$ is indexed by identical frequencies since coherence is again limited to same-frequency coupling. However, MIF is not limited to the linear Gaussian scenario, and remarkably can quantify statistical dependence in the frequency domain both for nonlinear and non-Gaussian situations.

In the previous subsection we mentioned the conditional extension of coherence which is well known as the partial coherence. However, a conditional extension of MIF which would allow for model-free partial frequency coupling analysis has yet to be introduced despite being suggested previously^7^. We note that prior work considered such a method with a Gaussian assumption^34^. We therefore introduce a model-free method, partial generalized coherence (PGC), as a partial expansion of MIF. With the consideration of a third random process *Z*(*t*), we define PGC as:

9 $$\begin{aligned} PGC_{XY|Z}(f_i,f_j|f_k) = I(d{\tilde{X}}(f_i)\,;\,d{\tilde{Y}}(f_j)\,|\,d{\tilde{Z}}(f_k)), \end{aligned}$$

which can be written as a difference of two MI terms^32^:

10 $$\begin{aligned} PGC_{XY|Z}(f_i,f_j|f_k) = I(d{\tilde{X}}(f_i)\,;\,d{\tilde{Y}}(f_j)\,,\,d{\tilde{Z}}(f_k)) - I(d{\tilde{X}}(f_i)\,;\,d{\tilde{Z}}(f_k)). \end{aligned}$$

Similar to what has been shown for MIF and coherence^7^, if *X*(*t*), *Y*(*t*), and *Z*(*t*) are all Gaussian processes, then we have a direct relationship between PGC and partial coherence (see Supplementary Information for proof based on prior work^34–37^):

11 $$\begin{aligned} PGC_{XY|Z}(f_i,f_i|f_i) = -\log (1-C_{XY|Z}(f_i)). \end{aligned}$$

However, the key contribution of our approach is the removal of model assumptions that limit partial coherence, as PGC quantifies partial frequency coupling for same-frequency and cross-frequency interactions. Furthermore, we will later introduce a new frequency coupling estimation procedure that scales better with dimensionality, enabling more spectral increments to be conditioned than previously possible.

Accordingly, it is important to state that our technique easily generalizes beyond conditioning on just one other spectral increment or process, and can even quantify the coupling between more than two spectral increments. A more general definition of PGC is then:

12 $$\begin{aligned} PGC_{XY|{\mathcal {Z}}}({\mathcal {F}}_i\,,\,{\mathcal {F}}_j\,|\,{\mathcal {F}}_k) = I(d{\tilde{X}}({\mathcal {F}}_i)\,;\,d{\tilde{Y}}({\mathcal {F}}_j)\,|\,{\mathcal {Z}}), \end{aligned}$$

where ${\mathcal {F}}$ indicates a set of frequencies, $d{\tilde{X}}({\mathcal {F}}_i)$ and $d{\tilde{Y}}({\mathcal {F}}_j)$ indicate the sets of spectral increments of *X* and *Y* for each frequency set ${\mathcal {F}}_i$ and ${\mathcal {F}}_j$, and ${\mathcal {Z}}$ indicates the set of spectral increments being conditioned for each frequency of set ${\mathcal {F}}_k$.

**Figure 2:**
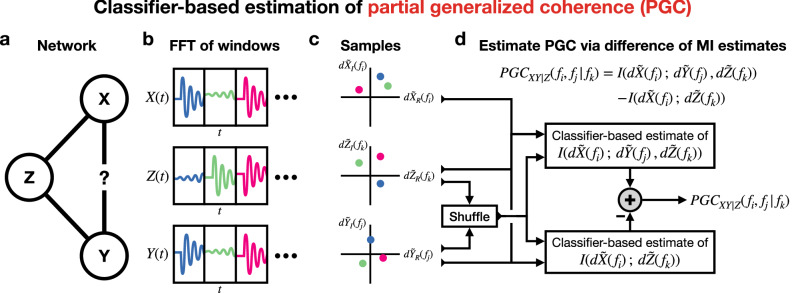
Overview of PGC estimation process. (**a**) Consider estimating the PGC between the processes *X* at $f_i$ and *Y* at $f_j$ conditioned on a third process *Z* at $f_k$. (**b**) Non-overlapping windows of the corresponding processes *X*(*t*), *Y*(*t*), and *Z*(*t*) are taken and then the FFT of each window is computed. (**c**) The FFT of each window of each process provides a complex sample of the spectral increment of interest. (**d**) Spectral increment samples are permuted to provide shuffled samples, and the degree to which a trained binary classifier can differentiate between the original and shuffled samples yields each MI estimate. Taking the difference of the two MI terms provides an estimate of PGC.

### Graph-based PGC

Because PGC quantifies conditional dependence between processes, one can easily extend it to define graphs where edges express partial frequency coupling as measured by PGC. In particular, we define a PGC graph *G* as:

13 $$\begin{aligned} G&= (V,E,w) \end{aligned}$$

14 $$\begin{aligned} V&= \{v^{(1)}, v^{(2)}, \dots , v^{(R)}\} \end{aligned}$$

15 $$\begin{aligned} E&= \{(x,y):x,y\in V\,\,\text {and}\,\,w^{(x,y)}>0 \} \end{aligned}$$

16 $$\begin{aligned} w^{(x,y)}&= PGC_{XY|{\mathcal {Z}}}({\mathcal {F}}_i\,,\,{\mathcal {F}}_j\,|\,{\mathcal {F}}_k) \end{aligned}$$

where *V* and *E* respectively denote the sets of vertices and edges while $w^{(x,y)}$ denotes the weight of an edge which is quantified by PGC. Therefore, the lack of an edge between two nodes would indicate that the two processes have no direct frequency coupling for frequency sets considered. Our simulations explore this concept for networks with three nodes, however it can be extended to networks of arbitrary size. Furthermore, PGC graphs can be embedded in a larger graphical analysis^38^.

### Estimating PGC

Whether estimating PGC for three processes or for a graph with many more processes the same overall procedure is used, which relies on the definition of PGC as a difference of individual MI terms (10) between spectral increments. The spectral increments $d{\tilde{X}}(f_i)$, $d{\tilde{Y}}(f_j)$, and $d{\tilde{Z}}(f_k)$ are continuous, and prior work^7^ compared the performance of two estimators of the MI between pairs of such vectors. One approach was kernel density estimation (KDE)^39^ which estimates probability distributions, while the other approach was *k*-nearest neighbors (*k*-nn)^40^ which estimates entropies. Although *k*-nn was found^7^ to be the better approach, both approaches are known to encounter issues when dimensionality is increased^17^. This is of great concern when estimating PGC because conditioning frequency coupling estimates on other nodes corresponds to increasing the dimensionality of the MI estimation problem. Therefore, an estimation approach that scales well with increasing dimensionality is needed to enable large scale partial frequency coupling analyses.

Recent work^17^ took a significantly different approach than that of KDE or *k*-nn by reframing MI estimation as a binary classification task. To understand the concept of this recent work^17^, consider that the original data of interest comes from a joint probability distribution which may contain dependence structures between spectral increments. The original data can be shuffled to produce samples which are effectively drawn from the independence distribution, i.e. the distribution obtained by taking the product of the marginal distributions of the spectral increments. If a trained binary classifier cannot identify a difference between samples from these two distributions^41,42^, then the spectral increments being considered are statistically independent and MI will be zero^17^. Therefore, the degree to which the classifier can differentiate between samples of these two distributions is quantified as MI. As demonstrated by the previous work^17^ and as shown in the results section of this current work, using a classifier approach to conditional MI estimation produces excellent scaling with dimensionality and significantly outperforms the scaling of *k*-nn.

Stating the classifier approach^17^ formally, consider that one has *N* samples of the original data which are drawn from the joint distribution $p_S$. This data can be shuffled to produce *N* samples of the independence distribution $q_S$, which is defined as the product of the marginal distributions. In order to use the samples from both of these distributions to estimate MI, consider the Donsker–Varadhan representation of the KL-divergence^17,43,44^:

17 $$\begin{aligned} D_{KL}(p_S\,||\,q_S) = \underset{f\in {\mathcal {F}}}{\sup }\bigg [\,\, \underset{s\sim p_S(s)}{\mathbb {E}}[f(s)] - \log \Big (\underset{s\sim q_S(s)}{\mathbb {E}}[\exp (f(s))]\Big )\bigg ], \end{aligned}$$

where ${\mathcal {F}}$ is a function class containing each function *f* yielding finite expectations. The representation in (17) becomes exact for the solution $f^*(s)=\log \frac{p_S(s)}{q_S(s)}$^17,43^, i.e. the point-wise log-likelihood ratio. Portions of the samples for the original data and the shuffled data can be used to train a binary classifier to approximate this ratio by labeling original samples as $\ell =1$ and shuffled samples as $\ell =0$^17,41,42^. The classifier then approximates $f^*$ by learning a likelihood function ${\mathcal {L}}(s_k) = \frac{P(\ell =1|s_k)}{1-P(\ell =1|s_k)}$ via the labeled training data, where $P(\ell =1|s_k)$ denotes the probability of the classifier identifying a sample $s_k$ as having come from the original data distribution^17^. This likelihood estimate can be plugged into (17) along with the use of averaging as an estimate of the expectation operator $\mathbb {E}$ to produce an estimate of the KL-divergence^17^:

18 $$\begin{aligned} {\hat{D}}_{KL}(p_S\,||q_S\,) = \frac{1}{N}\sum _{n=1}^{N}\log {\mathcal {L}}(s_{n,p}) - \log \Bigg ( \frac{1}{N}\sum _{m=1}^{N}{\mathcal {L}}(s_{m,q}) \Bigg ), \end{aligned}$$

where samples $s_{n,p}$ and $s_{m,p}$ are the remaining testing samples that were not used for training. Importantly, (18) is used to estimate each of the MI terms between spectral increments in (10) to produce an estimate of PGC.

However, we note that a bootstrapping process is used instead of relying on one single estimate of the KL-divergence. Specifically, considering the code for the previous work (https://github.com/sudiptodip15/CCMI)^17^, one bootstrap iteration consists of randomly selecting two-thirds of both the original data and the shuffled data for training the classifier while the remaining one-third is used as testing data, resulting in one estimate of the KL-divergence. Each additional bootstrap iteration again randomly splits the data into training and testing sets to produce more estimates. All of the estimates are averaged to produce one final estimate of the KL-divergence, and therefore increasing the number of bootstrap iterations improves the convergence of the final estimate. See the subsection titled “Baseline, control, & convergence analyses” for details on how convergence is verified empirically.

In the simplest case where PGC is being estimated between two spectral increments $d{\tilde{X}}(f_i)$ and $d{\tilde{Y}}(f_j)$ conditioned on a third increment $d{\tilde{Z}}(f_k)$ (Fig. 2a), there are two MI terms to estimate (10): $I(d{\tilde{X}}(f_i)\,;\,d{\tilde{Y}}(f_j)\,,\,d{\tilde{Z}}(f_k))$ and $I(d{\tilde{X}}(f_i)\,;\,d{\tilde{Z}}(f_k))$. One begins by taking non-overlapping windows of the three corresponding time series *X*(*t*), *Y*(*t*), and *Z*(*t*)^7^ (Fig. 2b). The FFT of each of these windows then provides individual samples $d{\tilde{x}}(f_i)$, $d{\tilde{y}}(f_j)$, and $d{\tilde{z}}(f_k)$ of the spectral increments $d{\tilde{X}}(f_i)$, $d{\tilde{Y}}(f_j)$, and $d{\tilde{Z}}(f_k)$^36^ (Fig. 2c). These spectral increment samples are referred to as the original samples, and then shuffled samples are generated by permuting the samples of $d{\tilde{Y}}(f_j)$ and $d{\tilde{Z}}(f_k)$^17^ while keeping the samples of $d{\tilde{X}}(f_i)$ ordered. To then estimate the first MI term $I(d{\tilde{X}}(f_i);\,d{\tilde{Y}}(f_j),\,d{\tilde{Z}}(f_k))$, (18) is used with $S=\{d{\tilde{X}}(f_i),\,d{\tilde{Y}}(f_j),\,d{\tilde{Z}}(f_k)\}$ meaning that the joint distribution is defined as $p_S = p_{d{\tilde{X}}(f_i),\,d{\tilde{Y}}(f_j),\,d{\tilde{Z}}(f_k)}$ and the independence distribution, i.e. what shuffled samples are drawn from, is defined as $q_S = p_{d{\tilde{X}}(f_i)}p_{d{\tilde{Y}}(f_j),\,d{\tilde{Z}}(f_k)}$. Portions of the original and shuffled samples are used to train a binary classifier with original samples having label $\ell =1$ and shuffled samples having label $\ell =0$, producing an estimated likelihood function ${\mathcal {L}}$. Plugging in this estimated function along with the remaining test samples $s_{n,p}$ and $s_{m,p}$ into (18) provides an estimate of $I(d{\tilde{X}}(f_i);\,d{\tilde{Y}}(f_j),\,d{\tilde{Z}}(f_k))$ (Fig. 2d), and multiple bootstrap iterations are used to provide an average estimate. Performing this overall process again with the omission of samples of $d{\tilde{Y}}(f_j)$ yields an estimate of the other MI term $I(d{\tilde{X}}(f_i);\,d{\tilde{Z}}(f_k))$, and the resulting difference between these two MI terms provides the PGC estimate (10) (Fig. 2d).

When the dimensionality of the PGC or MIF estimate is increased, such as including more spectral increments in conditioning for PGC, estimation bias will also increase and this is shown in the results subsection “Scaling analysis”. Accordingly, additional analyses were performed to account for this bias when comparing MIF and PGC estimates of different dimensionality. These additional control analyses along with analyses that produced baseline MIF values are detailed in the next subsection.

### Baseline, control, and convergence analyses

When needed, significance of results was evaluated via baseline MIF or control PGC analyses. Baseline MIF was computed between a signal at relevant frequencies and another signal at irrelevant frequencies. Such baseline values serve as a contrast to significant values resulting from the MIF between the same two signals both at relevant frequencies. Control PGC analyses between two signals involved conditioning on frequencies of a third signal that were known to be independent of the relationship between the first two signals. Control PGC analyses were therefore expected to produce PGC estimates bearing close resemblance to MIF estimates between the first two signals where nothing was conditioned, however control PGC values accounted for the increased estimation bias resulting from the dimensionality increase incurred by conditioning. Although quantifiable statistical significance could theoretically be pursued via non-parametric hypothesis testing, the computational complexity of the classifier approach makes this infeasible.

Because classifier-based estimates of MIF and PGC rely on bootstrapping, we also performed convergence analyses of estimates when studying the OB data. Considering that final estimates are averages across all bootstrap iterations, a cumulative average across bootstrap iterations was constructed. Then, the average of the 20 squared differences between the final 21 points of the cumulative average was computed. Importantly, the maximum average squared distance across all OB analyses was found to be 2.13e−6, meaning that all estimated frequency coupling values in the OB analysis strongly converged.

### Collection of calcium signals from OB glomeruli

All animal procedures were conducted in accordance with an animal protocol that was approved by the Institutional Animal Care and Use Committee (IACUC) of The University of Texas Health Science Center at Houston (UTHealth).

Calcium signals in OB glomeruli were recorded in an anesthetized naturally-breathing mouse. The experimental procedure was essentially the same as described previously^45^, except that calcium signals were recorded from glomeruli, which consist of neuropils rather than the cell bodies of neurons. Briefly, a progeny of Gad2-IRES-Cre^46^ (JAX stock #10802) and RCL-tdTomato^47^ (Ai9; JAX stock #7909) mice was used. The tdTomato marker was not utilized in this study. The mouse (male; 5 months old at recording) had received an injection of AAV vector (AAV1.Syn.GCaMP6f.WPRE.SV40; UPenn Vector Core) three weeks prior to the recording to express genetically-encoded calcium indicator GCaMP6f under the synapsin promoter. This resulted in the expression of GCaMP6f in multiple types of OB neurons, including mitral cells, tufted cells, periglomerular cells, and short-axon cells. For the recording, the mouse was anesthetized with urethane (6% w/v, 20 $\upmu$l/g bodyweight). Body temperature was maintained to 36–37 °C with a heat pad and the anesthetic was supplemented when the animal was responsive to a toe pinch. A cranial window was prepared above the dorsal OB and calcium signals were recorded with an acousto-optic deflector-based two-photon microscope equipped with a Nikon 16x/0.8NA objective lens. This two-photon microscope allows one to either record in the full-frame scan at 2 Hz or record in the random-access mode, in which signals are recorded only from pre-selected regions of interest (ROIs) at a much higher sampling rate. To define the ROIs, the odor-evoked responses to a few different odorants were recorded to identify the position of glomeruli. To assure that ROIs would not involve the cell bodies adjacent to the glomerulus, ROIs were set in the center part of each glomerulus. The sources of signal are dendrites of multiple types of OB neurons because of the specificity of the AAV vector used to express GCaMP. Within each of 10 identified glomeruli, two sites were chosen as ROIs, avoiding sub-areas with high-contrast. Calcium signals at the 20 defined ROIs were recorded at 500 Hz, without any odor presentation during recording. The breathing of the mouse was monitored via a piezo sensor attached to the rodent’s chest and recorded together with the calcium signal. 15 2-min blocks of ROI and breathing signals were used, providing 30 min of data in total. For each 2-min block, signals were z-scored.

## Results

We first analyze the functional connectivity in two simple three process simulations that highlight the impact of conditioning performed by PGC in frequency coupling analyses. Then, we show how the performance of PGC scales with higher dimensionality for two different estimation approaches. Finally, we analyze functional connectivity in the rodent OB by exploring how frequency coupling quantified by PGC is affected by controlling for breathing.

### Linear three process simulation

In order to provide intuition on what the conditioning ability of PGC achieves, we consider linear and nonlinear versions of a three process simulation. For the linear simulation, similar to a prior model^7^, the Gaussian random processes are defined as:

19 $$\begin{aligned} X(t)&= A_x\cos (2\pi f_0t+\Theta _x), \end{aligned}$$

20 $$\begin{aligned} W(t)&= X(t)+A_w\cos (2\pi f_0t+\Theta _w), \end{aligned}$$

21 $$\begin{aligned} Z(t)&= W(t)+A_z\cos (2\pi f_0t+\Theta _z), \end{aligned}$$

where $A_x$, $A_w$, and $A_z$ follow Rayleigh distributions with scale parameters $\sigma _x$, $\sigma _w$, and $\sigma _z$, respectively, while $\Theta _x$, $\Theta _w$, and $\Theta _z$ are each uniformly distributed from 0 to $2\pi$. All scale parameters were set to one, and 1e4 trials were simulated. Each trial was treated as a window which provided spectral samples via the FFT, meaning that estimates displayed for this simulation utilize 1e4 samples. Classifiers were trained using 100 bootstrap iterations.

**Figure 3:**
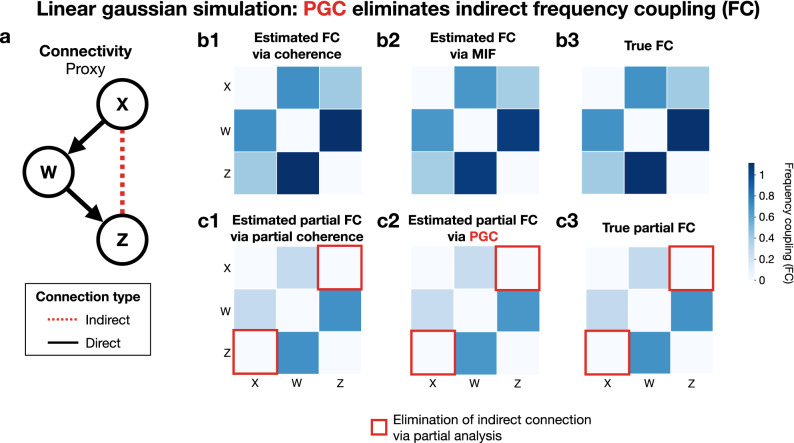
Linear interactions between simulated Gaussian processes. (**a**) Diagram of connectivity between processes *X*, *W*, and *Z* for $f_0$. This proxy configuration (Fig. 1a) induces an indirect connection between *X* and *Z*. (**b**) Pairwise analyses of same-frequency coupling between processes for frequency $f_0$. (**b1**) Frequency coupling (FC) estimate via transformed partial coherence (8). (**b2**) FC estimate via MIF (7). (**b3**) Analytical true FC, demonstrating FC estimation accuracy via coherence in (**b1**) and MIF in (**b2**). However, both pairwise methods ambiguously identify the indirect connection between *X* and *Z*. (**c**) Application of partial FC methods eliminates this indirect connection, revealing the direct connectivity structure. (**c1**) Estimated partial FC via transformed partial coherence (11). (**c2**) Estimated partial FC via PGC. (**c3**) Analytic partial FC, which demonstrates estimation accuracy of partial coherence in (**c1**) and PGC in (**c2**). Heatmaps generated via seaborn^48^.

As illustrated in Fig. 3a, the specified connectivity structure between these three processes produces an indirect relationship between *X*(*t*) and *Z*(*t*) which will be ambiguously included in pairwise analyses. To confirm this in simulations, the transformed coherence (8) and MIF (7) were estimated (Fig. 3b1,b2). Both detected the direct connectivity between *X* and *W* as well as between *W* and *Z*. However, both methods also included the indirect connection between *X* and *Z* as suspected. Additionally, the analytic value, referred to as the true frequency coupling (FC), was computed. Analytic values are known^7^ to be functions of the ratio of the relevant power spectral densities, which means that, for example, the FC between *X* and *W* is $\log \big ( 1 + (\sigma _x^2/\sigma _w^2)\big )$. Both coherence and MIF provided accurate estimates of the true FC values (Fig. 3b3).

The ambiguity of the direct or indirect nature of the detected connections requires the estimation of partial methods that condition pairwise analyses on other processes. Accordingly, the transformed partial coherence (11) and PGC were estimated (Fig. 3c1,c2), and the analytic partial FC was computed as well (Fig. 3c3). Similarly as before, partial coherence and PGC provide good estimates of the true partial FC (Fig. 3c3). The most notable feature of these estimates is the elimination of the indirect connection between *X* and *Z*, indicated by the white color in the off-diagonal corners of both heatmaps in Fig. 3c1,c2. This result illustrates the necessity of using partial analyses in identifying direct frequency coupling. Although both partial coherence and PGC were capable of eliminating the indirect connection in this linear Gaussian example, the capability of PGC to handle nonlinear and non-Gaussian processes is demonstrated in the next example.

A detailed comparison of estimation bias and variance for each technique is not pursued because the linear Gaussian case is a solved problem in that coherence and partial coherence are sufficient. Instead, these results are to show that MIF and PGC arrive at similar results compared to coherence and partial coherence in linear Gaussian models. The goal of PGC is to address the unsolved problem of partial frequency coupling in the nonlinear non-Gaussian case, which is demonstrated next.

### Nonlinear three process simulation

Neuronal activity is unlikely to be linear or Gaussian, and therefore we applied PGC to a more complex variation of the prior simulation to demonstrate the capability of PGC to handle the nonlinear and non-Gaussian scenario. The definition of *X*(*t*) remained unchanged (19) while the remaining processes were redefined to be:

22 $$\begin{aligned} W(t)&= X^2(t)+(A_w\cos (2\pi f_0t+\Theta _w))^2, \end{aligned}$$

23 $$\begin{aligned} Z(t)&= X^3(t)+(A_z\cos (2\pi f_0t+\Theta _z))^3, \end{aligned}$$

where as before $A_w$, and $A_z$ follow Rayleigh distributions with scale parameters $\sigma _w$, and $\sigma _z$, respectively, while $\Theta _w$, and $\Theta _z$ are each uniformly distributed from 0 to $2\pi$. Parameters $\sigma _w$ and $\sigma _z$ were set to 0.75. Relationships among processes are now between different frequencies, and this CFC is a result of *X*(*t*) being squared and cubed. Similar squaring was considered previously for pairwise MIF^7^. The presence of *X*(*t*) in the definitions of *W*(*t*) and *Z*(*t*) results in a common input motif (Fig. 1b) which is different from the prior proxy motif. 1e4 trials were simulated and 100 bootstrap iterations were used for MIF and PGC estimation.

**Figure 4:**
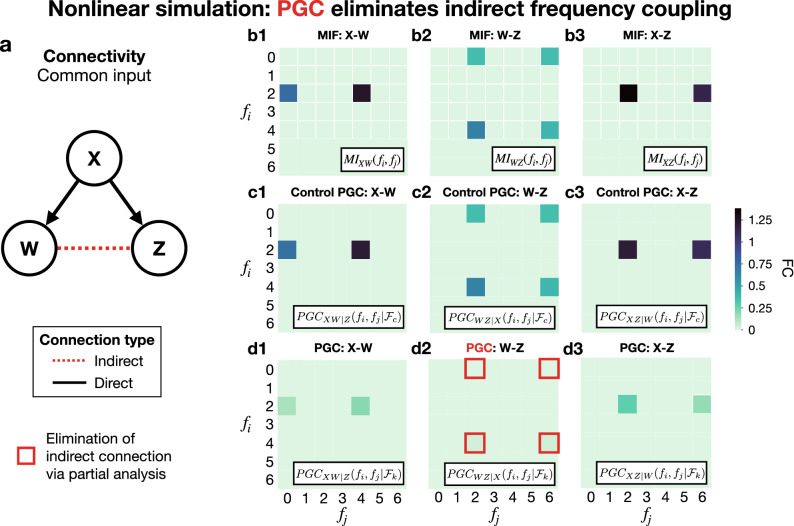
Nonlinear interactions between simulated processes resulting in direct and indirect coupling. (**a**) Diagram of cross-frequency coupling (CFC) between processes. Common input configuration (Fig. 1b) induces an indirect connection between *W* and *Z*. (**b**) Pairwise analyses of CFC between processes using MIF (7). (**b1**) MIF between *X* and *W*. (**b2**) MIF between *W* and *Z*, which ambiguously identified the indirect coupling for four different frequency pairings. (**b3**) MIF between *X* and *Z*. (**c**) PGC conditioned on irrelevant frequencies 1 and 3 Hz (denoted as the set ${\mathcal {F}}_c$) resulted in minimal changes. This analysis is referred to as control PGC, which serves as a contrast for the relevant conditioning performed in (**d**). (**c1**) Control PGC between *X* and *W*. (**c2**) Control PGC between *W* and *Z*. (**c3**) Control PGC between *X* and *Z*. (**d**) PGC was then conditioned on relevant frequencies, denoted as the set ${\mathcal {F}}_k$, of the other process for each pair of processes. (**d1**) PGC between *X* and *W*, which preserved the existence of direct coupling. (**d2**) PGC between *W* and *Z*, which resulted in the elimination of all indirect coupling between these two nodes. (**d3**) PGC between *X* and *Z*, which preserved the existence of direct coupling. Heatmaps generated via seaborn^48^.

Consider that the only non-zero spectral increment of *X*(*t*) is $d{\tilde{X}}(f_0)$. Since *W*(*t*) (22) now includes $X^2(t)$, the trigonometric identity $A\cos ^2(\theta )=\frac{A}{2}+\frac{A}{2}\cos (2\theta )$ makes it clear that *W*(*t*) will have non-zero spectral increments $d{\tilde{W}}(0)$ and $d{\tilde{W}}(f_0)$ that are both related to $d{\tilde{X}}(f_0)$. Similarly, the trigonometric identity $A\cos ^3(\theta )=\frac{3A}{4}\cos (\theta )+\frac{A}{4}\cos (3\theta )$ reveals that *Z*(*t*) will have non-zero spectral increments $d{\tilde{W}}(f_0)$ and $d{\tilde{W}}(3f_0)$ that are both related to $d{\tilde{X}}(f_0)$. The unintended consequence of this common input configuration is that *W*(*t*) and *Z*(*t*) will appear to have a relationship (Fig. 4a) that purely pairwise methods such as MIF cannot identify as indirect.

With $f_0=2$ Hz, MIF analysis of this simulation revealed the direct frequency coupling between *X*(*t*) and *W*(*t*) for $f_0$ and $\{ 0,2f_0 \}$ (Fig. 4b1) as well as the direct frequency coupling between *X*(*t*) and *Z*(*t*) for $f_0$ and $\{ f_0,3f_0 \}$ (Fig. 4b3). The four instances of indirect frequency coupling between *W*(*t*) and *Z*(*t*) for $\{ 0,2f_0 \}$ and $\{ f_0,3f_0 \}$ were also captured by MIF (Fig. 4b2). Importantly, there is no method for using MIF by itself to identify these four couplings as indirect.

Therefore, PGC is needed as before to address these indirect connections. In order to account for estimation bias that can arise from the increase in estimation dimensionality with PGC compared to MIF, we first considered the PGC between spectral increments was conditioned on the uninformative frequencies ${\mathcal {F}}_c$ of 1 Hz and 3 Hz. This PGC estimate is referred to as a control (see subsection titled “Baseline, control, & convergence analyses” for more detail) and serves as a contrast for when PGC is conditioned on frequencies with actual relevance to the estimation problem. When estimating the control PGC between *W* and *Z*, the uninformative frequency set ${\mathcal {F}}_c$ only included 1 Hz to account for the fact that *X* only has one non-zero spectral increment. The results of this control analysis are shown in Fig. 4c and confirm the intuition that conditioning PGC on unrelated spectral content should not change the resulting frequency coupling values in any dramatic way. However, conditioning PGC analyses on related spectral increments at frequencies ${\mathcal {F}}_k$ results in notably altered coupling estimates (Fig. 4d) compared to the control values (Fig. 4c). When estimating the PGC between the spectral increment of *X* at 2 Hz and the spectral increment of *W* at 4 Hz, for example, ${\mathcal {F}}_k$ will consist of 2 and 6 Hz because those are the frequencies of *Z* that were related to this pair of spectral increments. While the direct frequency coupling interactions were found to be appropriately still significant (Fig. 4d1,d3), PGC correctly eliminated the indirect frequency coupling between *W*(*t*) and *Z*(*t*) for all four frequency pairs (Fig. 4d2). Therefore, PGC is an effective tool for reducing indirect frequency coupling in the simple linear Gaussian case as well as the more complex nonlinear and non-Gaussian case.

**Figure 5:**
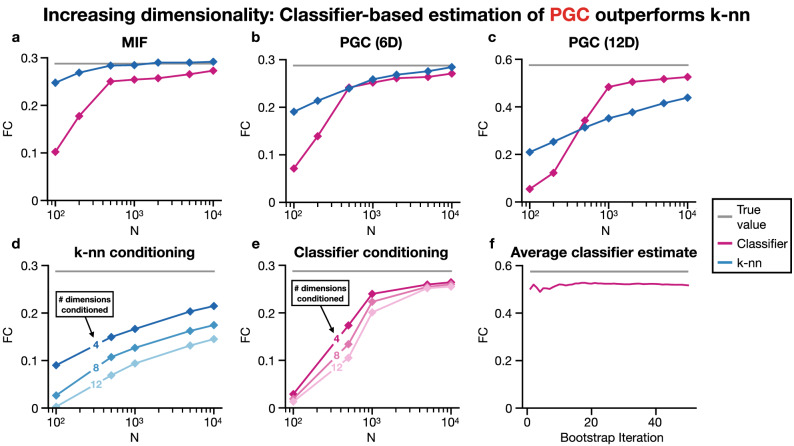
Estimating MIF and PGC between Gaussian spectral increments via *k*-nn and classifier. All plots except (f) include variance errorbars (too small to visually observe). (**a**) *k*-nn outperforms classifier for MIF, particularly for low samples. (**b**) *k*-nn has narrower advantage over classifier when estimating PGC between two spectral increments conditioned on a third increment (six-dimensional problem). (**c**) Classifier outperforms *k*-nn at $N=500$ samples and greater for larger twelve-dimensional problem where PGC is estimated between two pairs of spectral increments conditioned on a third pair of spectral increments. (**d**) Effect of conditioning *k*-nn-based PGC between two spectral increments on two pairs of spectral increments (four dimensions), four pairs of spectral increments (eight dimensions), and six pairs of spectral increments (twelve dimensions). (**e**) Analysis of classifier in same manner. Notably, with increasing sample size classifier significantly outperforms *k*-nn. (**f**) Using multiple bootstrap iterations for classifier-based estimation of PGC reduces estimate variability as shown by the cumulative average PGC estimate over bootstrap iterations.

### Scaling analysis

The ability of PGC to scale beyond three spectral increments is now considered in detail. For the purposes of scaling analyses, the spectral increments are considered to follow a multivariate Gaussian distribution. Therefore, the analytic MIF and PGC values are known based on covariance values and used for evaluating estimation performance. All scaling analyses (Fig. 5) used 100 independent simulations per plotted data point and 20 bootstrap iterations for each classifier-based estimate except for Fig. 5f which investigated performance over 50 bootstrap iterations for one simulation.

An important aspect of this analysis is the difference in performance between estimating PGC via classification, which has been used thus far in this manuscript, versus *k*-nn. In estimating the MIF between two spectral increments, which is a four-dimensional problem, *k*-nn appears to have a strong advantage in the low sample regime and a slight advantage in the remaining sample regimes when compared with the classifier approach (Fig. 5a). When considering the PGC between two spectral increments conditioned on a third spectral increment, which is a problem with six dimensions, the advantage of *k*-nn over the classifier approach narrows (Fig. 5b). Increasing the scale to a twelve dimensional problem (Fig. 5c) reveals that the classifier approach outperforms *k*-nn for higher dimensionality scenarios past a certain sample size ($N=500$), which is verified for other dimensionalities in Fig. 5d,e. The particular twelve dimensional problem was estimating the PGC between two pairs of spectral increments conditioned on another pair of spectral increments. The higher dimensionality cases in Fig. 5d,e were constructed by considering the PGC between two spectral increments conditioned on two pairs of spectral increments (four-dimensional conditioning), then conditioned on four pairs of spectral increments (eight-dimensional conditioning), and finally conditioned on six pairs of spectral increments (twelve-dimensional conditioning). Additionally, it is worth noting the effect of the number of bootstrap iterations on the classifier-based estimate of PGC. Increasing the number of bootstrap iterations reduces the variance of classifier-based PGC estimates (Fig. 5f), which should be kept in mind when performing PGC analyses.

Because the classifier approach outperforms *k*-nn for the analysis of the PGC between two pairs of spectral increments conditioned on another pair of spectral increments (Fig. 5c), it is used in analysis of the rodent OB which ultimately includes PGC estimation at the same scale of dimensionality. We also note that dealing with spectral leakage, which would involve including extra frequencies in PGC analysis where the signal has leaked into, was previously infeasible for certain sample ranges (Fig. 5) without the classifier approach.

### Glomerular calcium recordings from rodent OB

**Figure 6:**
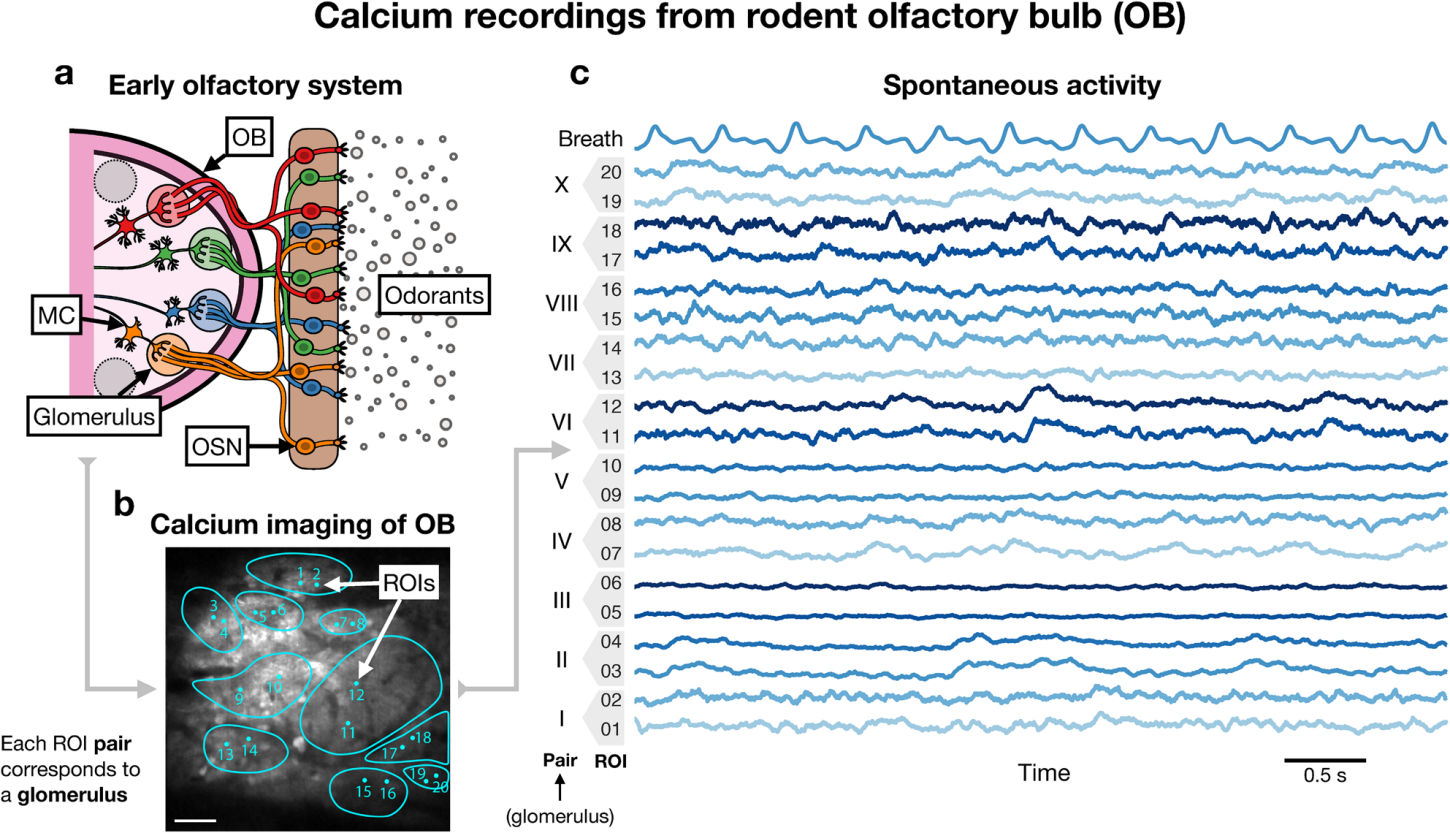
Data collection from rodent OB. (**a**) Odorants interact with olfactory sensory neurons (OSNs), which transmit activity to sites in the OB called glomeruli. Mitral cells (MCs) have dendrites in each glomerulus, and therefore MCs relay olfactory information to cortical areas for further processing. Connections exist between glomeruli but remain understudied. (**b**) GCaMP was used to perform two-photon imaging of glomeruli in the mouse OB. Pairs of ROIs were selected for each glomerulus which was identified based on odor responses and is indicated by a contour. Scale bar: 50 $\upmu$m. (**c**) Baseline activity, i.e. no odorant introduced, of each ROI along with a recording of a piezo sensor attached to the mouse’s chest to capture breathing activity.

Finally, we used classifier-based estimation of PGC to quantify the influence of breathing on glomerular relationships in the rodent OB^18^ for lower frequencies. The OB (Fig. 6a) is the fundamental structure of early olfactory processing. Olfactory sensory neurons (OSNs) respond to odorants, i.e. airborne molecules, and transmit their responses to particular sites in the OB called glomeruli. Other cells, mitral cells and tufted cells, then transmit the responses condensed at these sites to cortical areas for further olfactory processing which leads to odor perception. Although short-axon cells^19–21^ are known to enable inter-glomerular connectivity, relationships among glomeruli remain severely understudied. In estimating the functional connectivity between glomeruli, however, the influence of breathing is a potential problem because many OB neurons are known to driven by inhalation even without an odor presentation^22^.

In order to study such relationships, we utilized the genetically encoded calcium indicator GCaMP in the mouse OB (Fig. 6b). Pairs of ROIs were selected for each of the ten identified glomeruli (Fig. 6b). Breathing activity was additionally recorded by use of a piezo sensor attached to the mouse’s chest, and the trace is included at the top of Fig. 6c. All of the data displayed and considered in our analysis is baseline activity, i.e. recordings in the absence of a specific odorant stimulus, under anesthesia. In total, 30 min of data was collected at a sampling frequency of 500 Hz.

**Figure 7:**
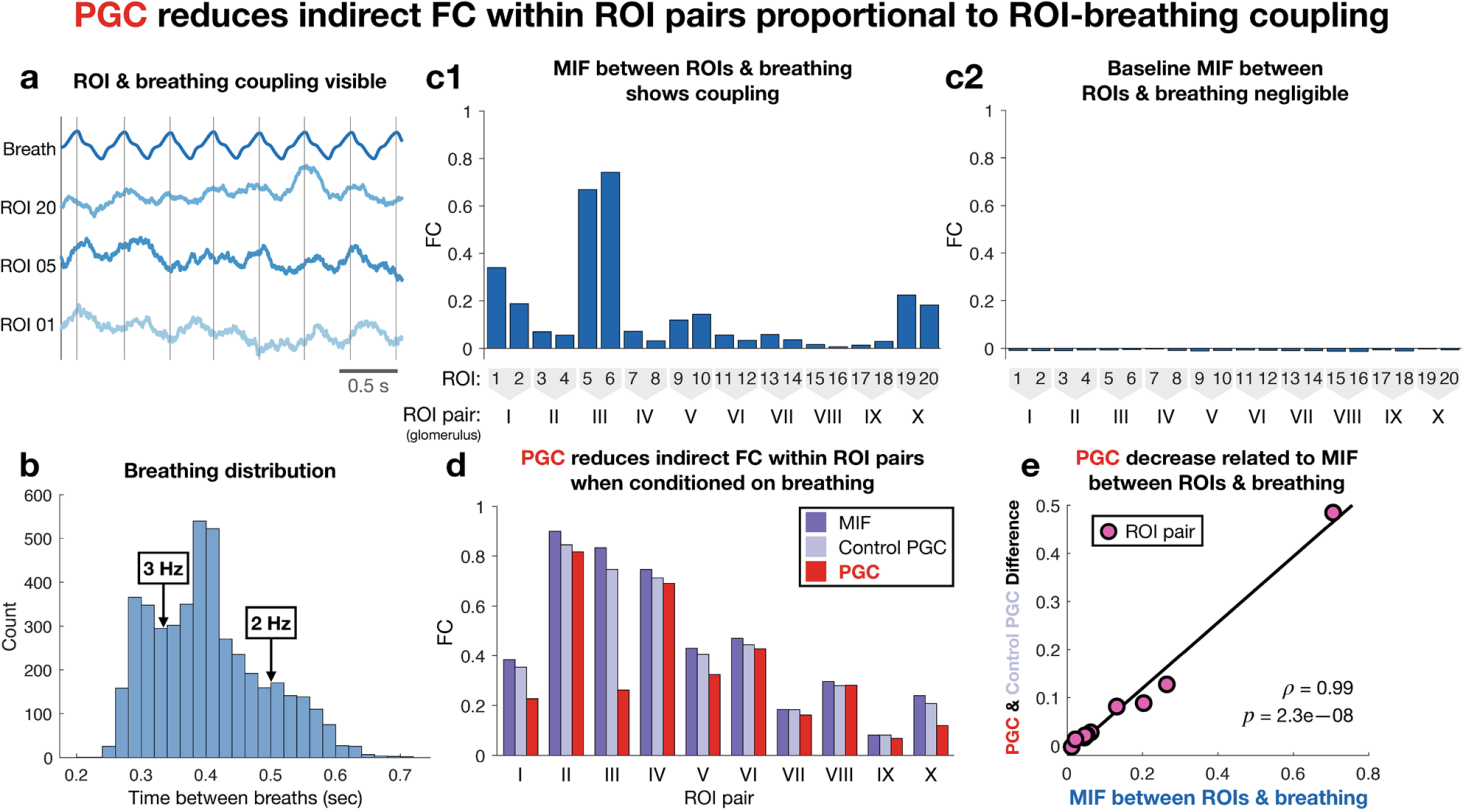
PGC analysis of ROIs and breathing. (**a**) Relationship between ROIs and breathing evident. Vertical lines indicate breathing waveform peaks. (**b**) Distribution of times between breaths suggesting 2 and 3 Hz are relevant frequencies for breathing. Times are differences between breathing peaks. (**c**) MIF between individual ROIs and breathing. (**c1**) MIF between individual ROIs and breathing for 2 and 3 Hz. (**c2**) MIF between individual ROIs (2 and 3 Hz) and breathing at irrelevant frequencies (49 and 50 Hz), which serves as a baseline. (**d**) First bar of each set is MIF between ROIs within pairs (same glomerulus) for 2 and 3 Hz. Second bar is PGC is computed between same ROIs for 2 and 3 Hz conditioned on two irrelevant frequencies of breathing (49 and 50 Hz) and is called control PGC. Third bar is PGC between ROIs for 2 and 3 Hz conditioned on 2 and 3 Hz of breathing. Conditioning PGC on 2 and 3 Hz of breathing usually resulted in decreased FC between ROIs compared to the control. (**e**) Visualization of linear relationship between ROI-breathing coupling (average of pair values in (**c1**)) and decrease in intra-pair coupling incurred by conditioning on breathing (control subtracted by PGC). Relationship had Pearson correlation coefficient $\rho =0.99$ and statistical significance $p=$ 2.3e−0.8 as computed by Matlab^49^ function corr.

The two aims of our analysis were to determine what functional connectivity exists in terms of frequency coupling between glomeruli for frequencies relevant to breathing and to what extent such connectivity can be reduced or eliminated via PGC by conditioning on the spectral components of the breathing waveform at these frequencies. Figure 7a makes it clear that the activities of some ROIs visually oscillate with breathing, which is notable since each pair of ROIs represents the activity of a glomerulus. In Fig. 7b we consider the distribution of time between breaths which is determined by taking the difference between peaks of the breathing waveform, which provides insight into what frequencies are relevant for breathing.

Taking 1 s windows of the recordings (Fig. 6c) provided 1800 samples of the spectral increments at each frequency of each channel with a frequency resolution of 1 Hz. Frequency coupling estimates were computed for frequencies 2 and 3 Hz because they are the most relevant according to the histogram in Fig. 7b. It should be noted that this type of analysis faces a trade-off between frequency resolution and the number of samples for each spectral increment. Using 2 and 3 Hz reasonably samples the set of breathing frequencies ranging from approximately 1.67–3.3 Hz (Fig. 7b). It is important to emphasize that the spectral samples for 2 and 3 Hz contain activity around both of these frequencies rather than only activity for exactly 2 and 3 Hz, which means our analysis accounted for breathing activity in general across this low frequency range. Furthermore, signal spectral leakage between 2 and 3 Hz will be included in these estimates since both frequencies are simultaneously used in FC estimates. 100 bootstrap iterations were used for training classifiers in all coupling estimates.

CFC between individual ROIs and breathing was first estimated via MIF for 2 and 3 Hz (Fig. 7c1), which for a given ROI *X* and breathing waveform *B* can be written mathematically as $MI_{XB}(\{2,3\},\{2,3\}) = I(d{\tilde{X}}(2),\,d{\tilde{X}}(3);\,d{\tilde{B}}(2),\,d{\tilde{B}}(3))$. Figure 7c1 reveals that some of the ROI time series have far greater coupling with breathing than others. In particular, ROI pairs I, III, V, and X have the strongest coupling with breathing. Coupling values for ROIs of the same pair tend to be similar, which follows from the fact that behavior across a glomerulus is generally homogeneous. As a baseline, MIF was computed between 2 and 3 Hz of the spectral increments of ROIs and irrelevant frequencies (49 and 50 Hz) of the breathing spectral increments, which confirmed that coupling in this control case was negligible (Fig. 7c2). Concern may arise from the displayed negative baseline MIF estimates (Fig. 7c2), however this non-zero result is simply due to the small bias of the displayed estimates. We further note that the magnitude of signal content is expected to be much lower than that of noise content at 49 and 50 Hz, and therefore these frequencies were chosen in order to provide a baseline.

**Figure 8:**
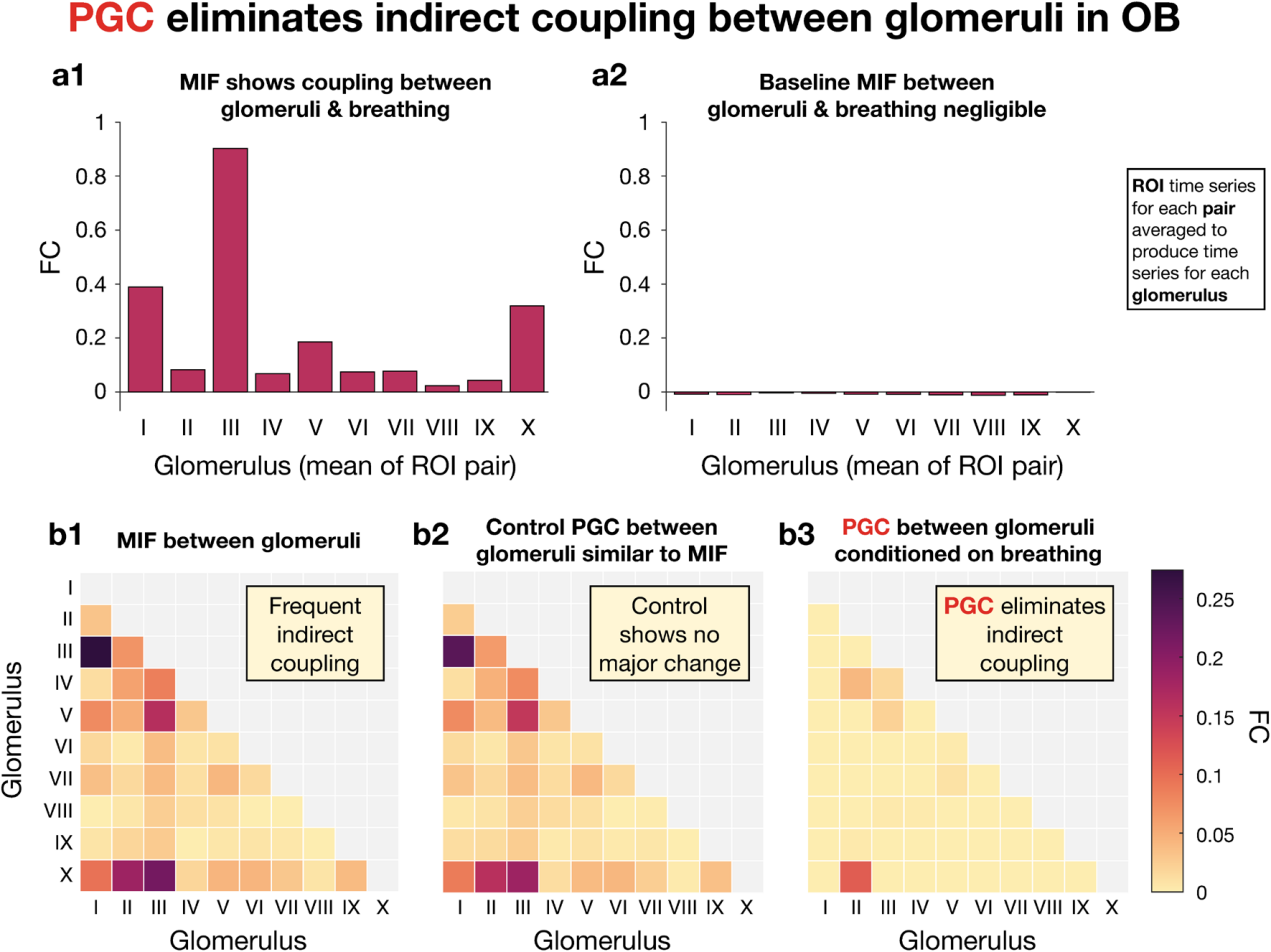
PGC analysis of glomeruli and breathing. (**a**): (**a1**) MIF between glomeruli (average of each ROI pair) and breathing for relevant frequencies 2 and 3 Hz. (**a2**) MIF between glomeruli (2 and 3 Hz) and breathing (49 and 50 Hz) to provide a baseline. Baseline MIF values were negligible because 49 and 50 Hz are uninformative frequencies of breathing. (**b**) FC between glomeruli. Diagonal and upper right grayed out because FC is symmetric. (**b1**) MIF between glomeruli for 2 and 3 Hz. (**b2**) PGC between glomeruli for 2 and 3 Hz conditioned on irrelevant breathing frequencies (49 and 50 Hz), referred to as control PGC. (**b3**) PGC between glomeruli for 2 and 3 Hz conditioned on breathing for 2 and 3 Hz. As expected, the control analysis (**b2**) yielded FC values nearly identical to those of not conditioning (**b1**). However, nearly all glomerular FC is eliminated when conditioning on breathing at relevant frequencies (**b3**), revealing almost all glomerular FC for 2 and 3 Hz to be indirect. Heatmaps generated via Matlab^49^.

Considering the results shown in Fig. 7c, we then sought to quantify the effect of breathing on coupling between ROIs in the same pair (i.e. same glomerulus). Accordingly, CFC was evaluated via the MIF between ROIs of the same pair for 2 and 3 Hz (dark purple bars of Fig. 7d). To determine how this intra-pair coupling is related to breathing, partial CFC was then evaluated via the PGC between ROIs conditioned on breathing. First, as a comparison, the PGC between ROIs for 2 and 3 Hz was conditioned on irrelevant breathing frequencies (49 and 50 Hz) and is referred to as control PGC (light purple bars of Fig. 7d). The control is important because it accounts for the increase in estimation bias that can arise from the increase in dimensionality incurred by conditioning. This bias is clear from the control values generally being slightly lower than the MIF values (Fig. 7d), however MIF and control values are still quite similar which follows from the fact that conditioning coupling analyses on irrelevant frequencies shouldn’t result in any significant changes apart from small changes in bias.

Comparing the control values with the PGC between ROIs for 2 and 3 Hz conditioned on 2 and 3 Hz of breathing (red bars of Fig. 7d) then reveals some remarkable drops in coupling between ROIs resulting from the removal of breathing effects. In particular, the decreases are the most marked for the aforementioned ROI pairs that had the greatest coupling with breathing, namely pairs I, III, V, and X. The decrease being greatest for these pairs is intuitive because they were the most coupled with breathing, and conditioning on breathing therefore removes a common input that accounted for a sizable portion of their intra-pair coupling. Figure 7e provides further evidence for this point by displaying these decreases against the previously computed ROI-breathing coupling (average of pair values in Fig. 7c1), demonstrating the strong linear relationship between both quantities.

Analysis culminated in the exploration of the effect of breathing on inter-glomerular relationships (Fig. 8). By averaging the time series for ROI pairs, time series were obtained for glomeruli because each pair corresponds to a glomerulus. The MIF between individual glomeruli and breathing activity was then estimated for 2 and 3 Hz (Fig. 8a1), which revealed that glomeruli I, III, V, and X had the strongest coupling with breathing. These glomeruli correspond to the same ROI pairs that had the highest coupling with breathing (Fig. 7c1). As a baseline, MIF was also estimated between glomeruli (2 and 3 Hz) and breathing for irrelevant frequencies (49 and 50 Hz), which appropriately produced near-zero coupling values (Fig. 8a2).

The CFC between glomeruli was then estimated via MIF for 2 and 3 Hz (Fig. 8b1), revealing many instances of coupling. Unsurprisingly, the strongest coupling instances tend to involve glomeruli I, III, V, and X. Before computing PGC with all relevant frequencies to demonstrate the indirect nature of these coupling instances, a control was produced (Fig. 8b2) by estimating the PGC between glomeruli (2 and 3 Hz) conditioned on breathing at irrelevant frequencies (49 and 50 Hz). Appropriately, the control (Fig. 8b2) is nearly identical to the non-partial coupling found previously (Fig. 8b1), which follows from the fact that nothing informative is being conditioned in the control. Comparing the control with the PGC between glomeruli (2 and 3 Hz) conditioned on relevant frequencies of breathing (2 and 3 Hz) reveals the dramatic extent to which glomerular coupling is indirect (Fig. 8b3). Importantly, PGC identified nearly all glomerular relationships for 2 and 3 Hz to be indirect, resulting in the sparse heatmap of Fig. 8b3. Even though non-negligible partial coupling still exists between glomeruli II and X, the partial coupling value has been markedly reduced by PGC in comparison to the control value. It is intuitive that the II–X relationship was not fully eliminated by conditioning on breathing, because glomerulus II did not exhibit a strong relationship with breathing (Fig. 8a1). The residual FC between glomeruli II and X potentially implies anatomical connectivity between them.

## Discussion

The distinction between direct and indirect frequency coupling is critical in neuroscience because of the insight afforded by knowing whether or not brain regions are directly functionally connected. Although partial coherence is sufficient for quantifying frequency coupling for Gaussian processes with linear relationships, many processes and relationships in the brain can be expected to be non-Gaussian and nonlinear. Importantly, no solution analogous to partial coherence has been introduced for performing partial frequency coupling analyses for such a complex case.

The aim of this work has therefore been to introduce PGC, a partial frequency coupling technique that does not make any model assumptions and that can condition on many other nodes. Although estimation of model-free frequency coupling has been achieved before via MIF^7^, the MI estimators used had limited ability to condition on other nodes due to poor scaling^17^ with the increased dimensionality necessary for such conditioning. By leveraging advances in the estimation of MI^17^, we have been able to formulate a partial coupling estimator that scales well even with conditioning on more than one node or even when considering the CFC among multiple frequencies simultaneously (Fig. 5).

We demonstrated the capability of PGC to address particular network motifs^23–25^ (Fig. 1). For the case where processes are Gaussian and interactions are linear, both partial coherence and PGC eliminated the indirect connection (Fig. 3) in a proxy configuration (Fig. 1a). Furthermore, estimates of both quantities verified the analytic relationship between partial coherence and PGC for the linear Gaussian case. PGC then correctly eliminated indirect connectivity in a common input case involving CFC resulting from nonlinear interactions between processes.

PGC was then applied to calcium data recorded from the rodent OB, revealing the practical nature of PGC. An important and understudied topic in olfactory research is the quantification of glomerular connectivity, which is known to exist anatomically but remains functionally undefined. The estimation of functional connectivity is a promising approach for answering this question, however a major issue is that breathing modulates glomerular activity^22^. PGC revealed that low frequency coupling between glomeruli is almost entirely attributable to the influence of breathing rather than any detectable influence between glomeruli. PGC is well-equipped to handle data outside of calcium imaging, and therefore we encourage the use of this technique on other types of continuously-valued recordings from the brain, such as ECoG and EEG. While our coupling analyses concerned elements within the rodent OB, application of PGC to larger scale recordings such as ECoG would allow for the determination of whether or not brain regions are directly coupled.

As suggested previously^7^, an interesting extension of this work would be wavelets. Wavelets provide a trade-off between temporal and spectral resolution that could be beneficial for time series analysis. One could also consider the use of other transform techniques, such as the chirp z-transform^50^ which offers improved spectral resolution within a narrower frequency range than afforded by the typical DFT. It would be additionally interesting to explore the performance of MIF and PGC when dealing with spectral leakage using the different estimation approaches available.

## Supplementary Information

Supplementary Information.

## Data Availability

The code for PGC is available on GitHub here: https://github.com/jy46/PGC (DOI: 10.5281/zenodo.4291286). The datasets generated during and/or analysed during the current study are available from the corresponding author on reasonable request.
