# Supplementary material for: Addressing indirect frequency coupling via partial generalized coherence
Source: Sci Rep. 2021 Mar 22;11:6535. doi: 10.1038/s41598-021-85677-6 (PMC7985302; doi:10.1038/s41598-021-85677-6)
Supplement: Supplementary file 1 — Supplementary Information. [file 41598_2021_85677_MOESM1_ESM.pdf]

Supplementary Information for:
Addressing indirect frequency coupling via partial generalized coherence

Joseph Young[1], Ryota Homma[2], and Behnaam Aazhang[1]

[1]Department of Electrical & Computer Engineering, Rice University, Houston, TX 77005, USA
[2]Department of Neurobiology & Anatomy, McGovern Medical School at the University of Texas Health Science Center at Houston, Houston, 77030, USA

Supplementary proof of relationship (11) between PGC and partial coherence

Here we prove the relationship (11) between PGC and partial coherence P for the linear Gaussian case, and omit f_i from all equations for readability. For the linear Gaussian case, PGC is equivalent to a difference in coherence terms [?, ?] (factor of $\frac{1}{2}$ removed as explained later):

$$PGC_{XY|Z} = -\log\left(1 - C_{X;YZ}\right) + \log\left(1 - C_{XZ}\right) \tag{S1}$$

$$= \log\left(\frac{1 - C_{XZ}}{1 - C_{X;YZ}}\right), \tag{S2}$$

where C_{XY} is the pairwise coherence considered in the main text, while $C_{X;YZ}$ is the multiple coherence defined as (page 296 of [?]):

$$C_{X;YZ} = \frac{1}{S_X}\begin{bmatrix} S_{XY} & S_{XZ} \end{bmatrix}\begin{bmatrix} S_Y & S_{YZ} \\ S_{ZY} & S_Z \end{bmatrix}^{-1}\begin{bmatrix} S_{YX} \\ S_{ZX} \end{bmatrix}. \tag{S3}$$

We note that prior works [?, ?] would include a factor of $\frac{1}{2}$ in (S2), which is appropriate when a vector of complex spectral increments, $d\tilde{X}$, $d\tilde{Y}$, and $d\tilde{Z}$, is used. By contrast, this work and the prior MIF work [?] use a larger vector, consisting of $d\tilde{X}_R$, $d\tilde{X}_I$, $d\tilde{Y}_R$, $d\tilde{Y}_I$, $d\tilde{Z}_R$, and $d\tilde{Z}_I$, where real and imaginary components are individual variables and the factor of $\frac{1}{2}$ will be absent. Because MI is a function of determinants of covariance matrices for the Gaussian case [?], the difference in covariance matrix determinants [?] between these two approaches accounts for the presence or absence of the $\frac{1}{2}$ factor.

Continuing the proof, the matrix inverse in (S3) is:

$$\begin{bmatrix} S_Y & S_{YZ} \\ S_{ZY} & S_Z \end{bmatrix}^{-1} = \frac{1}{S_Y S_Z - S_{YZ}S_{ZY}}\begin{bmatrix} S_Z & -S_{YZ} \\ -S_{ZY} & S_Y \end{bmatrix}, \tag{S4}$$

and its product with $\frac{1}{S_X}\begin{bmatrix} S_{XY} & S_{XZ} \end{bmatrix}$ is:

$$\frac{1}{S_X(S_Y S_Z - S_{YZ}S_{ZY})}\begin{bmatrix} S_{XY}S_Z - S_{XZ}S_{ZY} & -S_{XY}S_{YZ} + S_{XZ}S_Y \end{bmatrix}. \tag{S5}$$

The product of this result with $\begin{bmatrix} S^*_{XY} & S^*_{XZ} \end{bmatrix}^T$ is:

$$\frac{S^*_{XY}(S_{XY}S_Z - S_{XZ}S_{ZY}) + S^*_{XZ}(-S_{XY}S_{YZ} + S_{XZ}S_Y)}{S_X(S_Y S_Z - S_{YZ}S_{ZY})} \tag{S6}$$

$$= \frac{|S_{XY}|^2 S_Z - S^*_{XY}S_{XZ}S_{ZY} - S^*_{XZ}S_{XY}S_{YZ} + |S_{XZ}|^2 S_Y}{S_X(S_Y S_Z - |S_{YZ}|^2)}. \tag{S7}$$

Subtracting this result from 1 provides an expanded equation for the inner argument of the left term of (S2):

$$1 - C_{X;YZ} = \frac{S_X(S_Y S_Z - |S_{YZ}|^2) - (|S_{XY}|^2 S_Z - S^*_{XY} S_{XZ} S_{ZY} - S^*_{XZ} S_{XY} S_{YZ} + |S_{XZ}|^2 S_Y)}{S_X(S_Y S_Z - |S_{YZ}|^2)}. \tag{S8}$$

In order to acquire an expansion of the inner argument of the other term of (S2), consider:

$$1 - C_{XZ} = 1 - \frac{1}{S_X} S_{XZ} S_Z^{-1} S^*_{XZ} \tag{S9}$$

$$= 1 - \frac{|S_{XZ}|^2}{S_X S_Z} \tag{S10}$$

$$= \frac{S_X S_Z - |S_{XZ}|^2}{S_X S_Z}. \tag{S11}$$

Substituting the expanded denominator (S8) and numerator (S11) into PGC (S2):

$$PGC_{XY|Z} = \log\left(\frac{\dfrac{S_X S_Z - |S_{XZ}|^2}{S_X S_Z}}{\dfrac{S_X(S_Y S_Z - |S_{YZ}|^2) - (|S_{XY}|^2 S_Z - S^*_{XY} S_{XZ} S_{ZY} - S^*_{XZ} S_{XY} S_{YZ} + |S_{XZ}|^2 S_Y)}{S_X(S_Y S_Z - |S_{YZ}|^2)}}\right) \tag{S12}$$

$$= \log\left(\frac{\dfrac{S_X S_Z - |S_{XZ}|^2}{S_Z}}{\dfrac{S_X(S_Y S_Z - |S_{YZ}|^2) - (|S_{XY}|^2 S_Z - S^*_{XY} S_{XZ} S_{ZY} - S^*_{XZ} S_{XY} S_{YZ} + |S_{XZ}|^2 S_Y)}{(S_Y S_Z - |S_{YZ}|^2)}}\right) \tag{S13}$$

$$= \log\left(\frac{(S_X S_Z - |S_{XZ}|^2)(S_Y S_Z - |S_{YZ}|^2)}{S_Z S_X(S_Y S_Z - |S_{YZ}|^2) - |S_{XY}|^2 S_Z^2 + S^*_{XY} S_{XZ} S_{ZY} S_Z + S^*_{XZ} S_{XY} S_{YZ} S_Z - |S_{XZ}|^2 S_Y S_Z}\right) \tag{S14}$$

$$= \log\left(\frac{S_Y S_Z^2 S_X - |S_{XZ}|^2 S_Y S_Z - |S_{YZ}|^2 S_X S_Z + |S_{XZ}|^2 |S_{YZ}|^2}{S_X S_Y S_Z^2 - S_Z S_X |S_{YZ}|^2 - |S_{XY}|^2 S_Z^2 + S^*_{XY} S_{XZ} S_{ZY} S_Z + S^*_{XZ} S_{XY} S_{YZ} S_Z - |S_{XZ}|^2 S_Y S_Z}\right) \tag{S15}$$

$$= -\log\left(\frac{S_X S_Y S_Z^2 - S_Z S_X |S_{YZ}|^2 - |S_{XY}|^2 S_Z^2 + S^*_{XY} S_{XZ} S_{ZY} S_Z + S^*_{XZ} S_{XY} S_{YZ} S_Z - |S_{XZ}|^2 S_Y S_Z}{S_Y S_Z^2 S_X - |S_{XZ}|^2 S_Y S_Z - |S_{YZ}|^2 S_X S_Z + |S_{XZ}|^2 |S_{YZ}|^2}\right). \tag{S16}$$

Now consider that partial coherence can be defined as [**?**]:

$$C_{XY|Z} = \frac{|S_{XY|Z}|^2}{S_{X|Z} S_{Y|Z}}, \tag{S17}$$

where the residual power spectral density $S_{XY|Z}$ is [**?**]:

$$S_{XY|Z} = S_{XY} - \frac{S_{XZ} S_{ZY}}{S_Z} = \frac{S_Z S_{XY} - S_{XZ} S_{ZY}}{S_Z} \tag{S18}$$

$$= (S_Z S_{XY} - S_{XZ} S_{ZY}) S_Z^{-1}, \tag{S19}$$

and the residuals for X and Y are [?]:

$$S_{X|Z} = S_X - \frac{S_{XZ}S_{ZX}}{S_Z} = \frac{S_Z S_X - S_{XZ}S_{ZX}}{S_Z} \tag{S20}$$
$$= (S_Z S_X - S_{XZ}S_{ZX})S_Z^{-1} \tag{S21}$$
$$= (S_Z S_X - |S_{XZ}|^2)S_Z^{-1} \tag{S22}$$
$$S_{Y|Z} = S_Y - \frac{S_{YZ}S_{ZY}}{S_Z} = \frac{S_Z S_Y - S_{YZ}S_{ZY}}{S_Z} \tag{S23}$$
$$= (S_Z S_Y - S_{YZ}S_{ZY})S_Z^{-1} \tag{S24}$$
$$= (S_Z S_Y - |S_{YZ}|^2)S_Z^{-1}. \tag{S25}$$

The expanded residual power spectral densities can then be substituted into (S17):

$$C_{XY|Z} = \frac{S_{XY|Z}S^*_{XY|Z}}{S_{X|Z}S_{Y|Z}} \tag{S26}$$
$$= \frac{((S_Z S_{XY} - S_{XZ}S_{ZY})S_Z^{-1})((S_Z S_{XY} - S_{XZ}S_{ZY})S_Z^{-1})^*}{(S_Z S_X - |S_{XZ}|^2)S_Z^{-1}(S_Z S_Y - |S_{YZ}|^2)S_Z^{-1}} \tag{S27}$$
$$= \frac{(S_Z S_{XY} - S_{XZ}S_{ZY})(S_Z S_{XY} - S_{XZ}S_{ZY})^*}{(S_Z S_X - |S_{XZ}|^2)(S_Z S_Y - |S_{YZ}|^2)} \tag{S28}$$
$$= \frac{(S_Z S_{XY} - S_{XZ}S_{ZY})(S_Z S^*_{XY} - S^*_{XZ}S^*_{ZY})}{(S_Z S_X - |S_{XZ}|^2)(S_Z S_Y - |S_{YZ}|^2)} \tag{S29}$$
$$= \frac{S_Z^2|S_{XY}|^2 - S_{XZ}S_{ZY}S_Z S^*_{XY} - S^*_{XZ}S^*_{ZY}S_Z S_{XY} + |S_{XZ}|^2|S_{ZY}|^2}{(S_Z S_X - |S_{XZ}|^2)(S_Z S_Y - |S_{YZ}|^2)} \tag{S30}$$
$$= \frac{S_Z^2|S_{XY}|^2 - S_{XZ}S_{ZY}S_Z S^*_{XY} - S^*_{XZ}S^*_{ZY}S_Z S_{XY} + |S_{XZ}|^2|S_{ZY}|^2}{S_Z^2 S_X S_Y - |S_{XZ}|^2 S_Z S_Y - S_Z S_X|S_{YZ}|^2 + |S_{XZ}|^2|S_{YZ}|^2}. \tag{S31}$$

Subtracting this result from 1:

$$1 - C_{XY|Z} = 1 - \frac{S_Z^2|S_{XY}|^2 - S_{XZ}S_{ZY}S_Z S^*_{XY} - S^*_{XZ}S^*_{ZY}S_Z S_{XY} + |S_{XZ}|^2|S_{ZY}|^2}{S_Z^2 S_X S_Y - |S_{XZ}|^2 S_Z S_Y - S_Z S_X|S_{YZ}|^2 + |S_{XZ}|^2|S_{YZ}|^2} \tag{S32}$$
$$= \frac{S_Z^2 S_X S_Y - |S_{XZ}|^2 S_Z S_Y - S_Z S_X|S_{YZ}|^2 - (S_Z^2|S_{XY}|^2 - S_{XZ}S_{ZY}S_Z S^*_{XY} - S^*_{XZ}S^*_{ZY}S_Z S_{XY})}{S_Z^2 S_X S_Y - |S_{XZ}|^2 S_Z S_Y - S_Z S_X|S_{YZ}|^2 + |S_{XZ}|^2|S_{YZ}|^2} \tag{S33}$$
$$= \frac{S_Z^2 S_X S_Y - |S_{XZ}|^2 S_Z S_Y - S_Z S_X|S_{YZ}|^2 - S_Z^2|S_{XY}|^2 + S_{XZ}S_{ZY}S_Z S^*_{XY} + S^*_{XZ}S^*_{ZY}S_Z S_{XY}}{S_Z^2 S_X S_Y - |S_{XZ}|^2 S_Z S_Y - S_Z S_X|S_{YZ}|^2 + |S_{XZ}|^2|S_{YZ}|^2}. \tag{S34}$$

Taking $-\log$ of this result, rearranging, and using the fact that $S_{ZY} = S^*_{YZ}$ produces the following:

$$-\log(1 - C_{XY|Z}) \tag{S35}$$
$$= -\log\left(\frac{S_X S_Y S_Z^2 - S_Z S_X|S_{YZ}|^2 - |S_{XY}|^2 S_Z^2 + S^*_{XY}S_{XZ}S_{ZY}S_Z + S^*_{XZ}S_{XY}S_{YZ}S_Z - |S_{XZ}|^2 S_Y S_Z}{S_Y S_Z^2 S_X - |S_{XZ}|^2 S_Y S_Z - |S_{YZ}|^2 S_X S_Z + |S_{XZ}|^2|S_{YZ}|^2}\right), \tag{S36}$$

which is equivalent to (S16), proving that:

$$PGC_{XY|Z} = -\log(1 - C_{XY|Z}), \tag{S37}$$

which is the same as (11).
